# Women’s body odour during the ovulatory phase modulates testosterone and cortisol levels in men

**DOI:** 10.1371/journal.pone.0230838

**Published:** 2020-03-31

**Authors:** Wataru Tarumi, Kazuyuki Shinohara

**Affiliations:** Department of Neurobiology & Behavior Division of Advanced Preventive Medical Sciences Nagasaki University, Graduate School of Biomedical Sciences, Nagasaki City, Nagasaki, Japan; Pontificia Universidade Catolica do Rio Grande do Sul, BRAZIL

## Abstract

A growing body of evidence suggests that men may perceive women’s bodily odour to be more attractive during the high-fertility ovulatory phase than during other phases in the menstrual cycle. In particular, women’s bodily odour may influence important aspects of male mating behaviour, but the precise nature of this phenomena remains to be elucidated. Twenty-six men and five women participated in the study. Each woman wore a cotton T-shirt during the night for 3 days during the ovulatory phase, after which the regions of the T-shirt that had been in contact with the woman’s chest, armpits, and back, were cut out of the garment. We evaluated the changes in testosterone and cortisol levels in the saliva of men who smelled these cloth pieces. The odour emitted from the backs of women in the ovulatory phase was found to increase testosterone secretion in men, whereas the odour emitted from the chests of women in the ovulatory phase reduced cortisol secretion in men. These results suggest that the odour of specific body parts of women modulate unconscious physiological reactions in men.

## Introduction

Many mammals correctly discriminate their own odours from those of others, thereby not only defining their living and personal spaces but also accurately sensing the mating season to preserve their own species [1, 2]. For example, in male stump-tailed macaques (Macaca arctoides) exposed to vaginal secretions of females in the fertile period, there is an increase in the secretion of testosterone, a hormone that stimulates sexual desire [3]. Human males also detect the high-fertility (ovulatory) period in women by bodily odour [4], which may act as a form of sexual stimulant for men [5, 6]. Subjective evaluations by questionnaire have revealed that men prefer the bodily odour of women in the ovulatory phase to odour of women in the non-ovulatory phase [4, 7]. In addition, odours of the armpit and vulva of women in the ovulatory phase prompt an increase in testosterone levels and a decrease in cortisol levels in men who smell the odours [8, 9].

The functional odours of humans is secreted from the exocrine glands [10–13]. Exocrine glands include sebaceous glands as well as mammary glands, lacrimal glands and sweat glands such as apocrine glands [14]. Therefore, body areas that emit functional odours are not limited to the armpit and vulva. Testosterone levels in men decrease when they smell the tears of women experiencing negative emotions [15]. Human neonates are known to make a behavioural response to the pheromones contained in breast milk [16, 17]. There are also abundant sebaceous glands on the back and chest, and their secretory fluids contain steroid compounds [14]. Previous studies involving olfactory experiments in which subjects were exposed to steroid compounds suggested that the steroid compounds were involved in changes in human cognitive function and endocrine response [18–20]. Estratetraenol (EST), an estimated pheromone secreted by women, is one of the steroidal compounds [21,22]. EST helps men feel more feminine to women [21,22]. In addition, another study has shown that EST is related to men’s sexual arousal and sexual motivation [23,24]. Furthermore, EST may trigger change in men’s social cognition, especially in sexually related situations [25]. These reports remind us that there is a link between women’ back, chest odor and men’s sexual desire.

Although the functional roles of odour components derived from human exocrine glands remain unclear, the components are likely to be involved in sexual appeal to the opposite sex, mediated by olfaction [26, 27]. Therefore, identifying the sites where functional odours are secreted by women in the ovulatory phase is valuable for understanding behaviour related to sexual desire in men. We also focused on the odour of the upper body of women, such as the armpit, chest, and back in the present study. This is because human is easily exposed to the odor of the upper body due to the character of biped walking.

In summary, the odours of women in the ovulatory phase may stimulate the sexual desire of men by controlling endocrine responses in men who smell the odours. Such functional odours are likely to be emitted from the armpit, chest, and back, where exocrine glands are distributed. With this in mind, we investigated whether the functional odours of women in the ovulatory phase were emitted from the armpit, chest, and back.

## Materials and methods

### Participants

Thirty men (mean = 23.55 years, SD = 3.82) were enrolled in this study, but four men later changed their minds and dropped out. To prepare for the experiment, participants were asked to refrain from activities known to affect hormone levels: eating food or drinking caffeinated beverages or alcohol within 2 hours of testing, exercising within 12 hours of testing, or smoking within 6 hours of testing. All subjects provided written informed consent prior to their participation in this study. This research was approved by the ethics committee of Nagasaki University and executed according to the Declaration of Helsinki. IRB research approval number is 16012978.

### Odour collection

Five women (mean = 20.23 years, SD = 3.76) participated as scent donors. The women reported stable menstrual cycles, and had not used any form of hormonal contraception prior to their participation. We confirmed that there were no smokers among the donors or the donors’ immediate families. We provided luteinizing hormone surge detection kits (Rohto Pharmaceutical Co., Ltd., Osaka, Japan) to the donors as a tool to detect the timing of ovulation and confirmed when ovulation had occurred in each donor. The method of odour collection was based on previous research [8]. In brief, each donor wore a cotton T-shirt for 3 days from an ovulatory day at night while sleeping. We instructed the donor to place the shirt in a sealed bag each morning, store it in the refrigerator, and wear the same shirt for the 3 nights while sleeping. In order to exclude the possibility of odours of human life affecting the experimental results, we instructed donors to use unscented soap and shampoo when taking a shower every day and to refrain from using perfumes, deodorants, or antiperspirants, eating odorous food, drinking alcohol, using drugs, and having sex. After the 3 days, the donors returned the T-shirts to us, and we confirmed whether they had refrained from the aforementioned activities. All had adhered to the instructions. The T-shirt regions in contact with the chest, armpit, and back were cut into small pieces and stored in a freezer, where they remained when not in use.

### Odour smelling

After arriving at the laboratory, the male participants gargled with water and waited for 15 minutes for their mental state to stabilize. After that, we explained that male participants would smell the shirts worn by the women. The male participants were not informed as to which part of the shirt they would be smelling. Each participant was randomly assigned a T-shirt, and the small pieces of T-shirt that had been in contact with the chest, armpit, and back were each smelled by five or six men. To reduce the competing effect of the odour on the different pieces of T-shirt, participants smelled the odour of only one piece of T-shirt once each day, and this odour smelling was repeated for 3 days with the same participants. The method of odour smelling was based on previous research [8]. To obtain baseline hormone concentrations, the saliva of participants was collected by having them drool into a collection vial. Each participant was then instructed to place their nose into the opening of a plastic bag containing a piece of T-shirt and inhale three times. This odour smelling was repeated 5 and 10 minutes after the first inhalation. Fifteen minutes after the first inhalation, participants provided another saliva sample. Saliva samples were frozen at −80°C until assay.

### Hormone assay

Saliva samples were thawed completely and centrifuged at 1,500 g for 15 min at room temperature. The supernatant was used for measurement of testosterone levels. The hormone concentrations in the supernatant were measured with an ELISA kit (Salimetrics, State College, PA 16803, USA).

## Results

To investigate the effects of odour smelling on the testosterone and cortisol level of participants, the salivary these concentrations were entered into a two-way analysis of variance (ANOVA) with the within-participant factors of stimuli (chest—armpit—back) and Exposure Phase (Pre-Post) for the purpose of searching in an explorative manner for the cloth pieces that potentially modulate testosterone and cortisol secretion.

**Fig 1:**
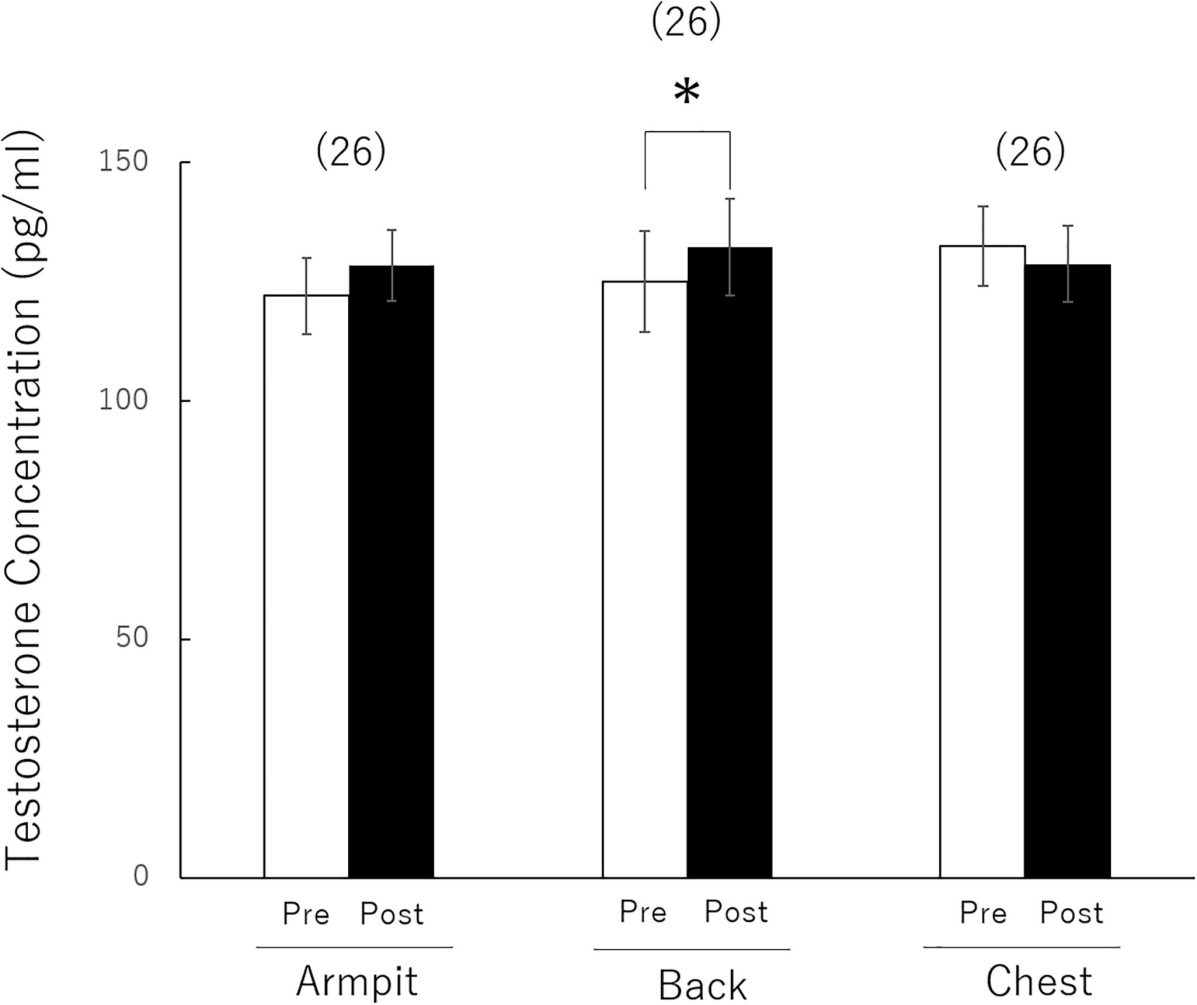
Comparisons of testosterone level changes to detect men’s response to odour of T-shirt regions in contact with the chest, armpit, and back in women with ovulatory phase. Data are expressed as the mean ± standard deviation. The number of participants in each group is given in parentheses. *P < 0.05.

An analysis of variance was performed for stimuli (F(2,50) = 0.249, P < 0.78, partial η2 = 0.009), Exposure Phase in testosterone level (F(1,25) = 2.974, P < 0.09, partial η2 = 0.106), and interaction of Exposure Phase in testosterone level and stimuli (F(2,50) = 4.902, P < 0.01, partial η2 = 0.164) (Fig 1). Analysis of the simple main effect of stimuli indicated a significant effect in the back group (F(1,75) = 6.867, P < 0.01). In addition, a pattern of increased testosterone levels was observed in armpit group ((F1,75) = 3.878, P < 0.05).

**Fig 2:**
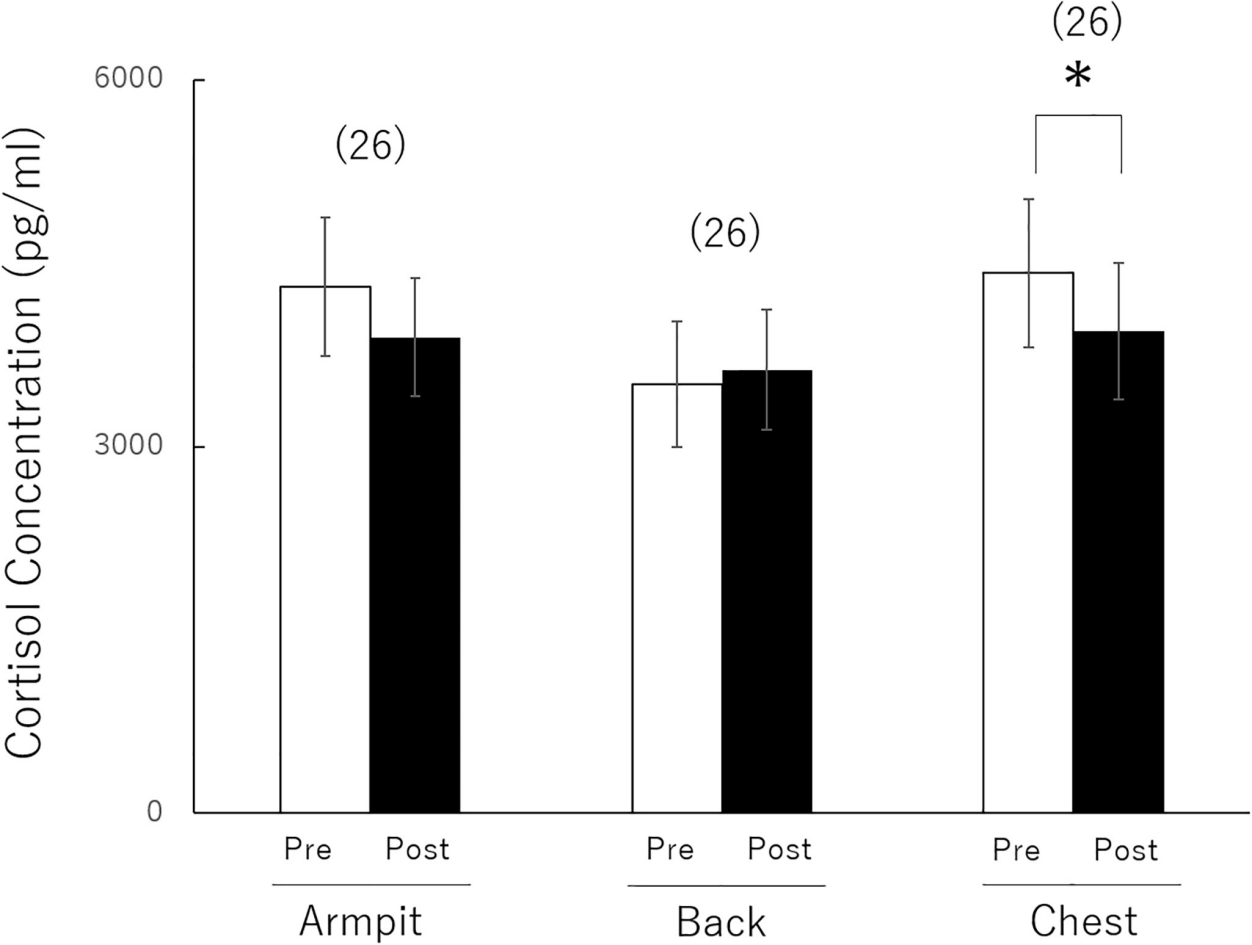
Comparisons of cortisol level changes to detect men’s response to odour of T-shirt regions in contact with the chest, armpit, and back in women with ovulatory phase. Data are expressed as the mean ± standard deviation. The number of participants in each group is given in parentheses. *P < 0.05.

To investigate the effects of odour smelling on the cortisol level of participants, using the same factors as in the case of testosterone, an analysis of variance was performed for stimuli ((F2,50) = 1.461, P < 0.24, partial η2 = 0.055), Exposure Phase in cortisol level ((F1,25) = 2.991, P < 0.09, partial η2 = 0.106), and interaction of Exposure Phase in cortisol level and stimuli (F(2,50) = 3.258, P < 0.04, partial η2 = 0.115) (Fig 2). Analysis of the simple main effect of stimuli indicated a significant effect in the chest group (F(1,75) = 5.156, P < 0.02).

## Discussion

The present study showed that functional odours producing physiological changes in men were emitted from the chests and backs of women in the ovulatory phase. The odour emitted from the backs of women in the ovulatory phase increased testosterone secretion in men. The odour emitted from the chest reduced cortisol secretion in men. Given that the ovulatory phase is a fertile period in female mammals, it would be natural to expect a form of unconscious communication between men and women in the ovulatory phase.

Men who smelled cloth pieces in contact with the back regions of women in the ovulatory phase displayed higher levels of testosterone in the post-exposure phase than in the pre-exposure phase. Conjecture based on the odour-emitting body parts of women in the ovulatory phase, and the physiological effect of such odours on men, suggests that the function of the odour of women in the ovulatory phase may be an evolutionary remnant derived from the mammalian mating posture. In nonhuman mammals, the odour of females in the ovulatory phase induces mating behaviour in males, and these animals have a common mating posture [28–32]. The female rodent mating posture is called lordosis: a male mounts a female from behind, during which the male is likely to be exposed to the odour of the female’s back. In other words, females may emit functional odours from their back during the ovulatory phase, and thereby stimulate male sexual desire by increasing testosterone secretion in males. However, it is not clear whether the odour emitted from the backs of females in the ovulatory phase induces male sexual desire in species other than humans. This hypothesis must be tested in future research.

Our results also suggest that the odour emitted from the chests of women in the ovulatory phase reduces cortisol secretion in men. During the neonatal period, human growth is generally based on drinking mothers’ breast milk, and a neonate and its mother likely communicate with each other through the smell of the breast milk [33, 34]. In addition, a neonate can discriminate its mother’s from others’ breast milk through smell [35, 36], the chest odour of lactating women is known to be attractive to neonates [37, 38], and cortisol levels related to stress induction in neonates can be suppressed by the odour of their mother’s breast milk [17, 39]. In light of these facts, the odour released from the chests of women may have an effect of decreasing cortisol levels related to stress. However, this hypothesis is based on the assumption that the odour components of breast milk and the chest are similar due to the proximity of the nipples and chest. Therefore, research that clearly distinguishes these effects must be conducted.

The present study demonstrated that odours which increased the secretion of testosterone in men were emitted from the backs of women in the ovulatory phase. In the present study, however, the influence of metabolites of steroid compounds, due to microorganisms, cannot be excluded; therefore, whether or not steroid compounds secreted from female sebaceous glands act directly on men remains unresolved. In the present study, a pattern of increased testosterone levels was also observed in men who were exposed to the armpit odour of women in the ovulatory phase, which is consistent with a previous study [9]. Therefore, the possibility of mixed odours from the armpit and back cannot be ruled out. Given this possibility, further investigation is required; however, our results suggest that functional odour components are emitted from the backs of women in the ovulatory phase.

Cortisol and testosterone are known to interact with each other to inhibit the hormonal effects of the other [40, 41]. The balance between these two hormones seems to regulate the expression of sexual desire and sexual activities [9, 42]. The present study showed that testosterone levels increased and cortisol levels decreased in men who had smelled the bodily odour of women in the ovulatory phase. Given that the bodily odour of women in the ovulatory phase sexually appeals to men, the study results are rational. This is because testosterone is associated with an increase in sexual desire in men and cortisol is related to a decrease in sexual desire in men. The results of this study revealed that the odours that increased the testosterone level and decreased the cortisol level were emitted from different parts of the body. Therefore, the effect of each odour may have been based on physiologically independent phenomena. However, we believe that women in the ovulatory phase blend two different odours to synthesize the odour that stimulates men’s sexual desire.

In summary, women in the ovulatory phase emit odours that increase testosterone levels and decrease cortisol levels in men; such functional odours are emitted from women’s chests and backs. In the present study, the same odorous clothing sample was provided to many subjects. Therefore, interactions between the subjects may have masked, or conversely enhanced, the effects of the odour. Environmental factors in the room where the experiment was conducted may also have affected the results. Finally, the action mechanism of the odour perceived by the subjects has not been elucidated. Although further study is required to resolve these questions, our results suggest that odours modulate unconscious communication between men and women.
